# Supplementary material for: Anticancer Effect of E26 Transformation-Specific Homologous Factor through the Induction of Senescence and the Inhibition of Epithelial–Mesenchymal Transition in Triple-Negative Breast Cancer Cells
Source: Cancers (Basel). 2023 Nov 2;15(21):5270. doi: 10.3390/cancers15215270 (PMC10650711; doi:10.3390/cancers15215270)
Supplement: Supplementary file 1 [file cancers-15-05270-s001.zip › cancers-2673747-supplementary.pdf]

cancers

MDPI

Article

Anticancer Effect of E26 Transformation-Specific Homologous Factor through the Induction of Senescence and the Inhibition of Epithelial–Mesenchymal Transition in Triple-Negative Breast Cancer Cells

Soyoung Lim, Jihyun Lim, Aram Lee, Keun-Il Kim and Jong-Seok Lim

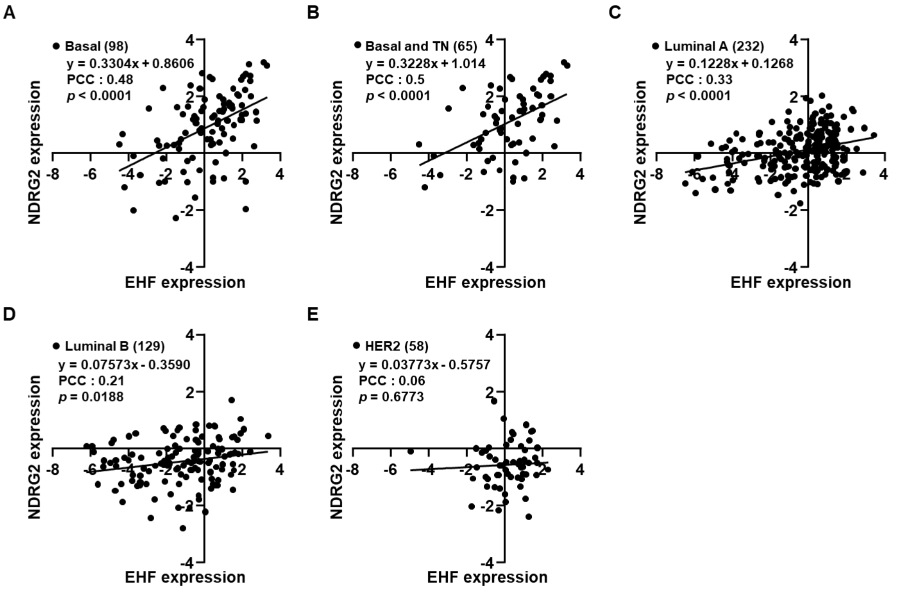

Figure S1. NDRG2 and EHF expression levels were positively correlated in the basal subtype of breast cancer. (A-E) Correlation graph of NDRG2 and EHF expression in TCGA breast cancer patients in basal (A), basal and TN (B), luminal A (C), luminal B (D), and HER2 (E) subtypes.

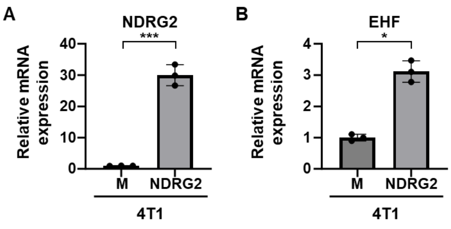

Figure S2. High expression of EHF in 4T1-NDRG2 cells. Increased EHF expression in 4T1-NDRG2 cells. (A, B) RT–qPCR data showing the relative expression of NDRG2 and EHF in 4T1-mock and 4T1-NDRG2 cells. *p<0.05, ***p<0.001.

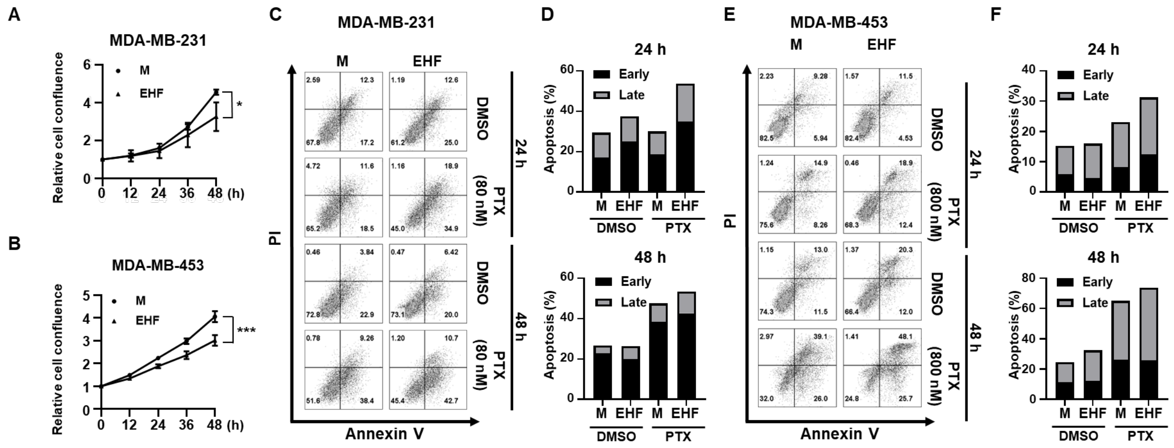

Figure S3. EHF expression suppressed cell growth and promoted paclitaxel-induced apoptosis. (A) The relative cell confluence of MDA-MB-231 cells was calculated using IncuCyte ZOOM® after transfection of pCMV-Taq2B (mock) (circle symbol) and pCMV6-Entry-EHF (EHF) (triangle symbol). (B) The relative cell confluence of MDA-MB-453 cells was calculated using IncuCyte ZOOM® after transfection with pCMV-Taq2B (mock) (circle symbol) and pCMV6-Entry-EHF (EHF) (triangle symbol). (C) MDA-MB-231 cells were treated with DMSO or 80 nM of paclitaxel for 24 or 48 hours after transfection of pCMV-Taq2B (mock) and pCMV6-Entry-EHF (EHF). Cells were stained for Annexin V/PI analysis. (D) Early and late apoptosis rates of MDA-MB-231-mock and -MDA-MB-231-EHF cells. (E) MDA-MB-453 cells were treated with DMSO or 800 nM of paclitaxel after transfection with pCMV-Taq2B (mock) and pCMV6-Entry-EHF (EHF) for 24 or 48 hours. Cells were stained for Annexin V/PI analysis. (F) Early and late apoptosis rates of MDA-MB-453-mock and -MDA-MB-453-EHF cells. *p<0.05, ***p<0.001.

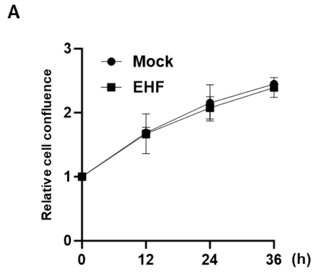

Figure S4. Mitomycin C treatment attenuated the growth of MDA-MB-231-mock and MDA-MB-231-EHF cells. (A) MDA-MB-231-mock (circle symbol) and -MDA-MB-231-EHF cells (square symbol) were treated with 20 μg/ml and 2.5 μg/ml mitomycin C, respectively. After 4 hours of treatment, the relative cell confluence was calculated by IncuCyte ZOOM®.

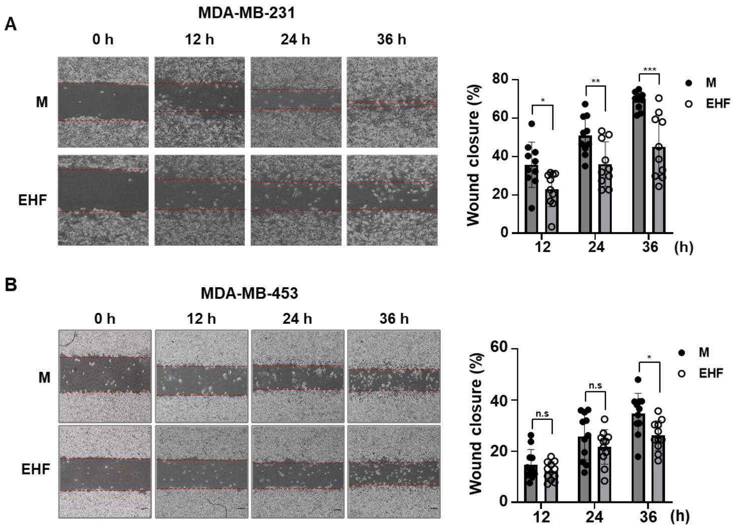

Figure S5. EHF expression inhibited the migration of TNBC cells. (A, B) Representative images of wound healing analysis after transfection of pCMV-Taq2B (mock) and pCMV6-Entry-EHF (EHF) in MDA-MB-231 (A) and MDA-MB-453 (B) cells. The red dotted lines indicate the borders of the wound at 0, 12, 24, and 36 hours after scratching. The wound closure percentage was calculated as the ratio of the remaining gap at each time point to the original scratch gap at 0 h. *p<0.05, **p<0.01, ***p<0.001.

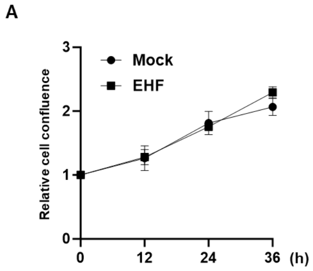

Figure S6. Mitomycin C treatment attenuated the growth of 4T1-mock and 4T1-EHF cells. (A) The 4T1-mock (circle symbol) and 4T1-EHF cells (square symbol) were treated with mitomycin C at 10 μg/ml and 2.5 μg/ml, respectively. After 4 hours of treatment, the relative cell confluence was calculated by IncuCyte ZOOM®.

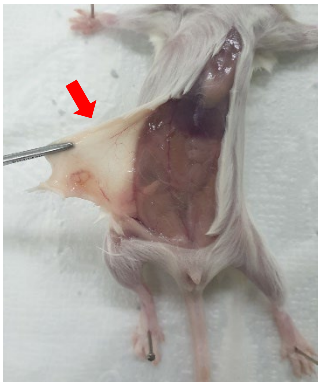

Figure S7. Complete tumor regression in 4T1-EHF injected mouse. The 4T1-EHF cells were subcutaneously injected into five Balb/c mice and two of them showed complete tumor regression (the injection site is indicated by an arrow).

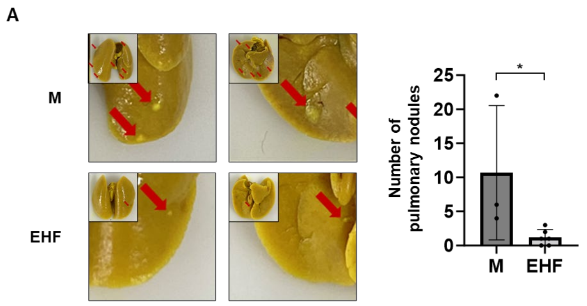

Figure S8. EHF overexpression in 4T1 mouse tumor cells suppressed pulmonary nodule formation in vivo. (A) Representative image of the lungs of Balb/c mice injected subcutaneously with 4T1-mock and 4T1-EHF cells (left). A graph showing the number of pulmonary nodules (right). *p<0.05.

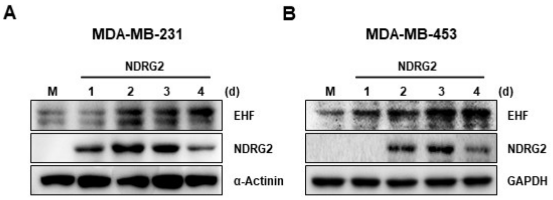

Figure S9. Overexpression of NDRG2 induced EHF expression in MDA-MB-231 and MDA-MB-453 cells. The cells were harvested at indicated time after overexpression of NDRG2 in MDA-MB-231 cells (A) and MDA-MB-453 cells (B).

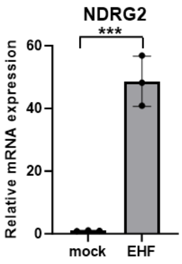

Figure S10. Stable overexpression of EHF induced NDRG2 expression in MDA-MB-231 cells. The relative mRNA expression of NDRG2 were measured by RT–qPCR in MDA-MB-231-mock and MDA-MB-231-EHF cells. *** $p < 0.001$.

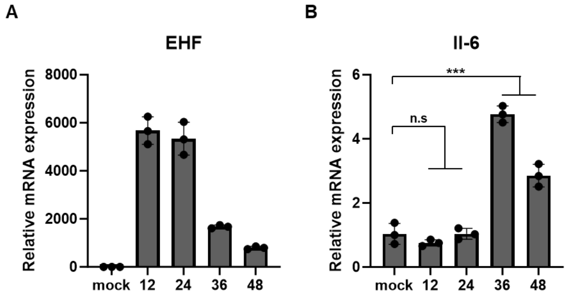

Figure S11. Overexpression of EHF induced IL-6 mRNA expression in MDA-MB-453 cells. The relative mRNA expression of IL-6 after transient overexpression of EHF was measured in MDA-MB-453 cells. *** $p < 0.001$.
